# Stimulatory Functions of Male Genitalia in *Tipula* (*Triplicitipula*) *colei* Alexander and *Tipula* (*Lunatipula*) *translucida* Doane (Diptera: Tipulidae) and Implications for Theories of Genital Evolution

**DOI:** 10.3390/insects15090680

**Published:** 2024-09-09

**Authors:** William G. Eberhard, Jon K. Gelhaus

**Affiliations:** 1 Smithsonian Tropical Research Institute and Escuela de Biología, Universidad de Costa Rica, Ciudad Universitaria, Costa Rica, and Museum of Natural Science, Louisiana State University, Baton Rouge, LA 70808, USA; 2 Department of Biodiversity, Earth and Environmental Sciences, The Academy of Natural Sciences, Drexel University, 1900 Ben Franklin Parkway, Philadelphia, PA 19103-1195, USA; jkg78@drexel.edu

**Keywords:** sexual selection, genital behavior, rapid divergent evolution, species isolation, lock-and-key, sexually antagonistic coevolution, cryptic female choice

## Abstract

**Simple Summary:**

The morphological designs and the behavior (rhythmic brushing, vibrating, scraping, and tapping) of the male genitalia in two species of crane flies indicate that male genital structures in both species function to stimulate the female during copulation. These observations are used to test current theoretical explanations of the rapid divergent evolution of male genitalia of animals with internal insemination.

**Abstract:**

Male genitalia have been hypothesized to function as courtship devices during copulation, but it is difficult to use behavioral observations to test this hypothesis because male genitalia are usually hidden inside the female during copulation. In tipuloid flies, however, nearly all of the male’s complex genital structures remain outside the female. Copulation behavior and genital morphology in *Tipula* (*Triplicitipula*) *colei* and *T*. (*Lunatipula*) *translucida* suggest that some male genital structures function to stimulate the female: male structures that contact the female bear tufts or dense arrays of modified setae on precisely the surfaces that contact the female; contact involves repeated, stereotyped rhythmic movements that include brushing, vibrating, scraping, and tapping; the movements are appropriately designed to utilize the morphology of the modified setae to stimulate the female; and the movements have little or no other perceptible mechanical effects on the female. The female structures contacted by these male genital movements fail to show the defensive designs predicted by the theories of genital evolution that are based on morphological species isolation or male–female morphological conflicts of interest; also unexplained by the conflict of interest hypothesis are female movements that seem designed to increase rather than avoid stimulation by the male.

## 1. Introduction

One of the most consistent trends in the evolution of animal species with internal insemination is for genital structures, especially those of males, to diverge more rapidly than other morphological traits [1]. The cause of this widespread evolutionary pattern is controversial. There is general accord that the “common sense” function of male genitalia, to transfer sperm from the male to the female, is unable to explain this evolutionary diversity, but there are several alternative explanations. Currently popular hypotheses emphasize post-copulatory sexual selection, including sperm competition, cryptic female choice, and sexually antagonistic coevolution [2,3,4,5,6]. An older species isolation hypothesis (“lock-and-key”) is also sometimes cited [7,8], despite extensive evidence against its general applicability [1,3,9,10].

Testing these hypotheses has been difficult, partly because the male’s genitalia are usually hidden inside the female where their behavior during copulation cannot be observed. In addition, the morphology of the portions of the female’s reproductive tract that are contacted by the male genitalia are also often hidden and difficult to observe [11,12]. Thus, it is generally not feasible to use the classic technique of combining observations of the behavior and the physical properties of structures to test hypotheses regarding their functions [13]. Most studies of the functional morphology of intromittent genitalia have been limited, at best, to “snapshots” of behavior provided by flash-frozen pairs (e.g., [14,15,16,17,18]). 

Flies in the family Tipulidae offer a partial escape from these limitations. Despite their ungainly long legs and often erratic flight, tipulids comprise one of the most speciose families in Diptera, with approximately 4357 recognized species [19]. The superfamily Tipuloidea, which includes Tipulidae, is an ancient group and may represent (along with Trichoceridae) the sister taxon of nearly all other Diptera [20,21,22]. The genitalia of tipulids fit the typical pattern: they are complex, divergent, and usually species-specific in form, and the genitalia of males tend to be more diverse and species-specific than those of females (e.g., [21,23,24,25,26,27,28]). The setae on the male genitalia are also diverse in form, and some are quite elaborate (e.g., [27]). 

Importantly, the elaborate genitalia of male tipulids remain largely on the outer surface of the female during copulation, where their behavior can be observed [21,25,26,29,30,31,32,33,34,35]. Only a thin, unadorned tube-like intromittent organ is inserted into the female’s body, where sperm are stored in three spermathecae [36]. The female structures with which the male’s genitalia interact are on her outer surface, so they can also be easily observed. In sum, the powerful combination of using behavior and morphological design to understand functional morphology [13] can be employed to explore the evolution of tipulid genitalia. 

This study employs video recordings to obtain especially detailed observations of the copulation behavior of two species, *Tipula* (*Triplicitipula*) *colei* Alexander 1942 and *T*. (*Lunatipula*) *translucida* Doane 1901, whose behavior has not been studied previously. The basic objective is to test the hypothesis that male genital structures function to stimulate females by checking for behavioral and morphological traits whose designs suggest this function. The data have major theoretical consequences because several hypotheses regarding genital evolution, including species isolation, sexually antagonistic male–female coevolution, and cryptic female choice, have alternative versions, one that emphasizes male stimulation and one that emphasizes male mechanical fit and force.

## 2. Materials and Methods

Still photos and video recordings (at 30 fps) were made using an Olympus “Tough” TG-4 camera (obtained from Amazon.com in Baton Rouge, LA, USA) of a copulating pair of *T.* (*T.*) *colei* after the unrestrained flies were placed under a dissecting microscope. The flies were found in copula at 9:00 P.M. on March 16, 2024 in residential Baton Rouge, LA (elevation 15 m, latitude 30.409416, longitude −91.154519), and they separated spontaneously approximately 3 h later. Still photos were taken of two pairs of *T*. (*L*.) *translucida* in a meadow near Cullowhee NC (el. 646 m, latitude 35.313709, longitude −83.176537) on 20 May, 2024, using a Nikon D850 with portable lights and a macro lens, and a third pair was video-recorded (at 60 fps) with the same camera the next day for the last 2:20 min of copulation before the pair separated. The male’s genital behavior was also recorded during the subsequent 2:30 min, documenting movements that revealed possible additional forces that the male genitalia may exercise on the female during copulation. 

The positions of most male and female genital structures were determined by direct observation, but the positions of a few that were hidden from view were deduced by their locations with respect to nearby structures in non-copulating males and females; all such indirect conclusions are specified in the descriptions. Some setae were only visible when illuminated from particular directions and are thus not visible in some photos. Unless noted otherwise, all mentions of dorsal vs. ventral and anterior vs. posterior orientations in the descriptions of copulating animals are with respect to the female. In some places, we use the descriptive term “stalk” of Hemmingsen [26,32] to avoid using the clumsy phrase “the portion of the female’s abdomen covered by and including tergites 9 and 10”. We follow Frommer [36] in the complex terminology of tipulid genital structures unless noted otherwise. Means are followed by ± one standard deviation. Voucher specimens are deposited in the Arthropod Museum of Louisiana State University.

## 3. Results

### 3.1. Genital Morphology and Behavior of T. (T.) colei

#### 3.1.1. Male Morphology

**Figure 1:**
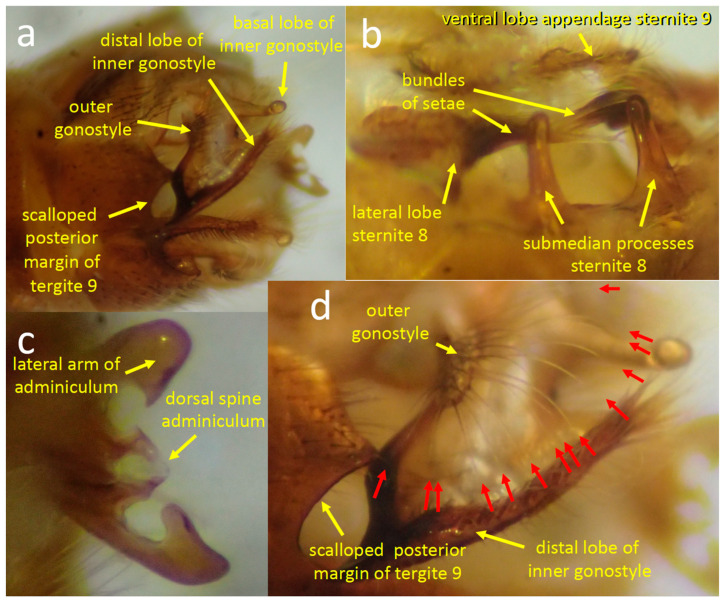
Male genitalia of *Tipula* (*Triplicitipula*) *colei*. (**a**) The scalloped distal edge of tergite 9, the outer gonostyle, andthe outer basal lobe and distal lobe (crest) of the inner gonostyle. (**b**) Each lateral lobe of sternite 9 bears a bundle of long, stiff setae projecting medially. The surface between the submedian processes is smooth and each process has a rounded tip. The flat, setose ventral lobes of the appendage of sternite 9. (**c**) The distal tip of the adminiculum; the central dorsal spine has a groove (arrow) through which the intromittent organ presumably passed during copulation and the distal edges of its two lateral arms form a curved surface that pressed against the anterior wall of the female’s genital chamber (see Figure 3). (**d**) The long setae projecting beyond the edge of the outer gonostyle are indicated with red arrows.

**Figure 2:**
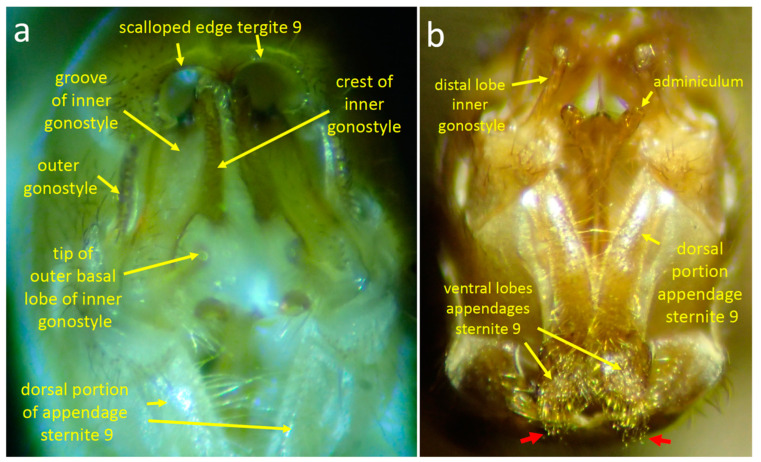
Male genitalia of *Tipula* (*Triplicitipula*) *colei* in posterior views. (**a**) This somewhat dorsal-posterior view shows the scalloped posterior edge of tergite 9, the inner and outer gonostyles, and the basal portion of the appendage of sternite 9. (**b**) A more ventral-posterior view shows the dorsal portion and the plate-like ventral portion of the sternite 9 appendage (red arrows indicate long setae on the ventral portion) and the adminiculum.

The posterior margin of sternite 8 bears a pair of ventrally projecting lateral lobes, and the tip of each lobe bears a rod-like bundle of long, stiff, medially directed setae (Figure 1b). A pole-like submedian process with a rounded tip is just anterior and medial to each lateral lobe (Figure 1b). The surface of sternite 8 between the submedian processes is smooth and curves slightly ventrally near the base of each process (Figure 1b). Each of the pair of long ventral appendages of the male’s sternum 9, called A9S (Figure 2a,b), bears at its tip a more or less rounded, slightly cupped lobe (Figure 2b) with abundant long setae on its distal edge (red arrows in Figure 2b).

There is a pair of complexly sculptured inner and outer gonostyles just posterior to the scalloped posterior margin of tergite 9 (Figure 1a,d and Figure 2a). The lateral border of each inner gonostyle bears a reinforced crest that borders a deep groove that opens anteriorly (Figure 2a). The cuticle is darker at the anterior end of the groove, forming an extension called a beak. The base of the curved, planar outer gonostyle (Figure 1a,d and Figure 2a) articulates near the base of the inner gonostyle. The anterior edge of the outer gonostyle bears a row of anteriorly projecting long, gently curved yellow setae (red arrows in Figure 1d) in addition to the short, dark setae on other portions of its outer surface. The posterior edge of tergite 9 just anterior to the gonostyles has a pair of deep indentations (Figure 1a,d and Figure 2a).

The adminiculum, a long, thin, stiff extension of the intersegmental membrane of the male, forms a hollow extension of the male’s outer surface [36]. The distal tip of the adminiculum has a pair of lateral lobes whose curved distal margins give its distal edge a crescent shape (Figure 1c and Figure 2b). The male’s thread-like intromittent organ passes through the adminiculum and exits distally via its central dorsal spine (Figure 1c).

#### 3.1.2. Female Morphology

**Figure 3:**
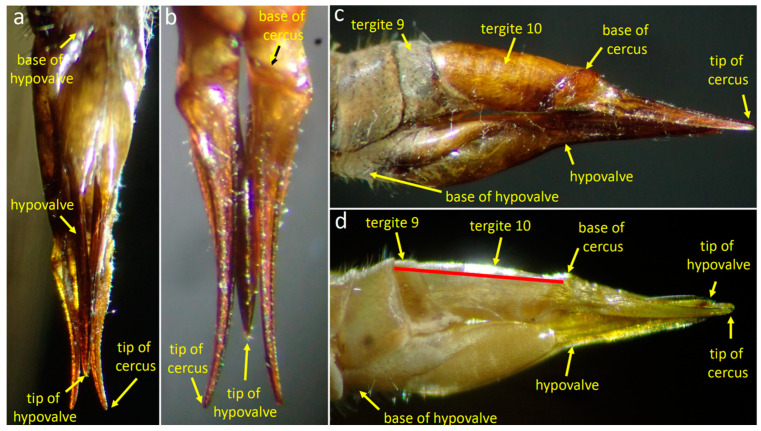
Female genitalia of *Tipula* (*Triplicitipula*) *colei* in ventral (**a**), dorsal (**b**), and lateral (**c**,**d**) views. The basal portion of the hypovalve is thick and highly sculptured, while its flattened, pointed tip extends posteriorly almost to the tips of the cerci. The cuticle is more transparent in (**d**) and the approximate extent of the “stalk” of Hemmingsen is marked with a red line.

The dorsal side of the posterior end of the female abdomen is formed by modified tergites 9 and 10 (the “stalk” of [32]) (red line in Figure 3d), and a pair of mobile cerci that articulate basally at the distal edge of tergite 10 (Figure 3b); the distal portions of the cerci curve laterally (Figure 3a,b). The forms of the cerci and the sculpturing on their lateral surfaces (Figure 3b) suggest that they dig forcefully in a substrate by opening laterally during oviposition, as in some other tipulids [37]. The ventral side of the abdomen bears a pair of long, blade-like hypogynial valves or hypovalves (Figure 3a–d) that are extensions of sternum 8 [36] (Frommer 1963) and also presumably function during oviposition [37]. 

**Figure 4:**
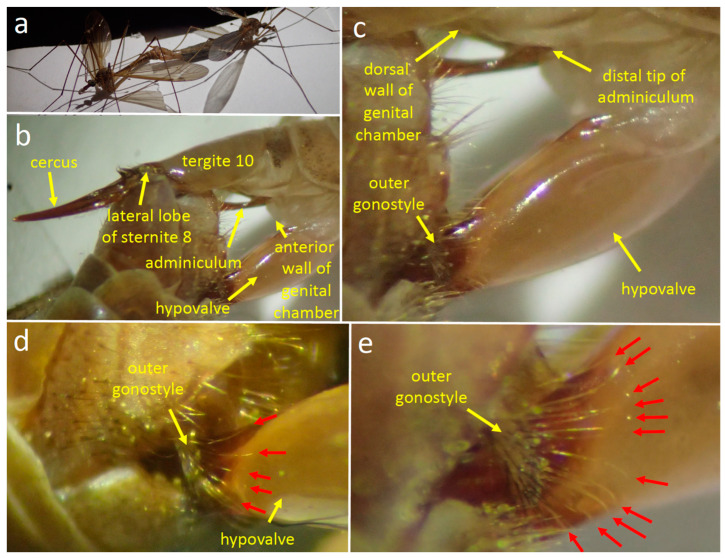
Copulation in *Tipula* (*Triplicitipula*) *colei.* (**a**) Male (left) and female (right) resting on a piece of paper. (**b**,**c**) The female’s tergite 10 and hypovalve are held apart by the male’s genitalia, while the male’s adminiculum presses against the anterior wall of her genital chamber. (**d**,**e**). The long setae extending from the anterior edge of the male’s outer gonostyle (red arrows) rest on the distal surface of the female hypovalve. A more nearly complete array is visible in (**e**) than in (**d**) due to favorable lighting.

The genital chamber, where the egg resides temporarily after it has emerged from the oviduct during oviposition [37,38], is bounded below by the canoe-like form of the basal portion of the hypovalves, above by the ventral membrane of the stalk, and anteriorly by the intersegmental membrane (Figure 4b) where the egg emerges from the oviduct through the gonopore [36]. 

#### 3.1.3. Positions of the Genitalia during Copulation

The flies were calm enough that the positions of their genitalia changed little and could be observed during the entire 3 h during which copulation was observed. As in other tipulids [21,32], the male’s abdomen was twisted 180° (Figure 4a) so that his sternites contacted dorsal female structures and his tergites contacted ventral female structures. In general terms, the male’s genitalia spread the female’s cerci and hypovalves apart, exposing her gonopore in the anterior wall of her genital chamber (Figure 4b). On the female’s ventral side, the tips of her hypovalves pressed against the male’s dorsal surface (Figure 4b–e); each tip was probably lodged in the deep groove of the male’s inner gonostyle, but this detail was not observed directly. The long yellow setae along the posterior edge of the male’s outer gonostyle splayed across the smooth lateral surface of the female’s hypovalve near its distal end (red arrows in Figure 4d,e). 

The curved distal tip of the male’s adminiculum pressed against the membranous anterior wall of the female’s genital chamber (Figure 4c). A small portion of the thin, dark, hair-like aedeagus [36] or “intromittent organ” [27] was visible where it passed through the dorsal spine of the adminiculum and (presumably) through the female gonopore into her common oviduct (this detail was not observed directly). The intromittent organ was the only male structure that was topologically inside the female. The scalloped rear margin of male tergite 9 was not observed directly, but its position relative to the female suggests that it did not contact her.

**Figure 5:**
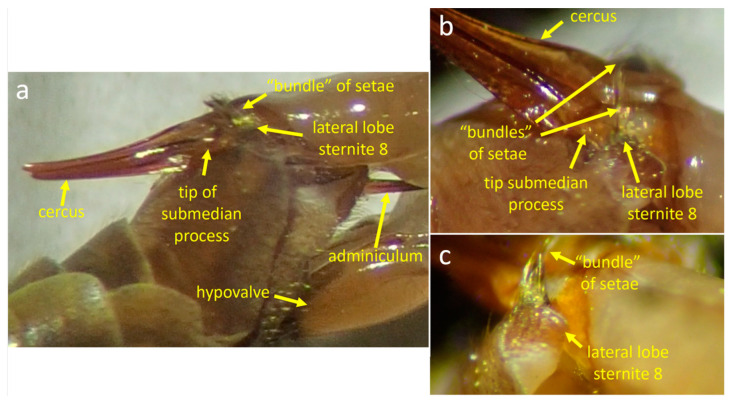
Lateral views of the lateral lobe of male sternite 8 pressing against the female of *T*. (*T*.) *colei*. (**a**) The bundle of long, stiff setae of the lateral lobe apparently contacts the dorsal surface of the bases of the cerci. (**b**) The lateral lobe and the submedian process are visible near the membranous articulation of a cercus with the female’s tergite 10. (**c**) The long setae are apparently dorsal to the bases of the cerci.

**Figure 6:**
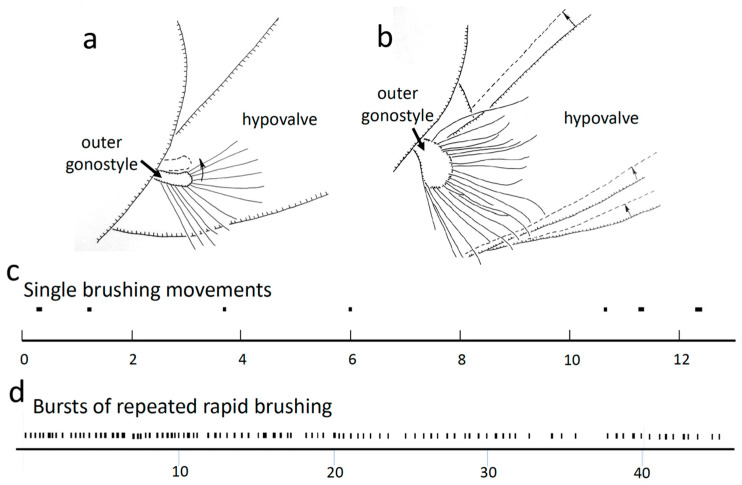
Diagrammatic illustrations of movements in *T*. (*T*.) *colei* of the long setae on the male’s outer dististyle brushing the surface of the female’s hypovalve traced from video recordings (**a**,**b**). (**a**) The brushing movement on the female’s hypovalve was produced by movement of the male’s outer gonostyle (the arrow indicates the position of the outer gonostyle 0.03s later). (**b**) The movement of the gonostyle setae was produced by movement of the female hypovalve (dotted lines indicate positions 0.13 s later). (**c**) Temporal pattern of single brushing movements of the outer gonostyle that immediately preceded squeezes by the female (black bars represent time spent moving). (**d**) Temporal pattern of bursts of rapid gonostyle brushing movements (time units in the graphs are seconds).

Each of the lateral lobes of the male’s sternite 8 pressed against the membranous indentation in the female’s lateral surface where her cercus articulated with tergite 10 (Figure 4b); they may have thus prevented her abdomen from sliding posteriorly or anteriorly with respect to the male. At some moments the submedian process of his sternite 8 also appeared to press either into this groove or just posterior to it (Figure 5a,b). The bundle of long stiff setae of the male’s lateral lobes that projected medially were at some moments in the air just dorsal to surfaces of her cerci (Figure 5c), but at other moments they appeared to contact the cerci’s dorsal surfaces or the intersegmental membrane near their bases (Figure 5a and Figure 6). The ventral lobes of the male’s appendages on sternite 9 and the brushes of long setae along their distal edges probably pressed against the dorsal wall of her genital chamber, but their positions were not observed directly.

#### 3.1.4. Movements of Male Structures That Contacted the Female during Copulation

Adminiculum

**Figure 7:**
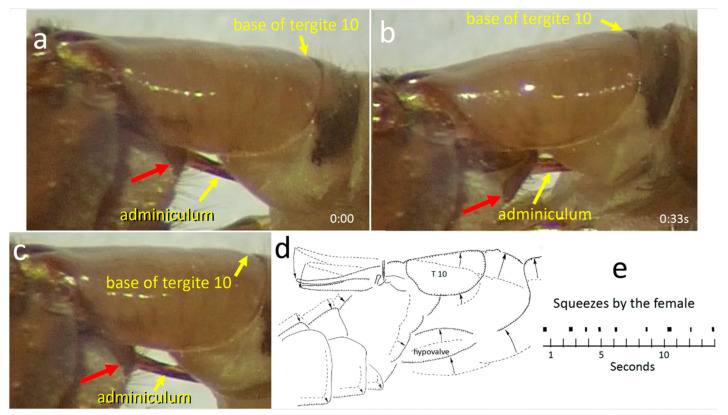
(**a**–**c**) Frames of a video recording of a squeeze in a dorso-lateral view of *T*. (*T*.) *colei* (**a**–**c**) illustrate how the adminiculum and the base of the female’s tergite 10 moved dorsally in a period of 0.47 s (the red arrows indicate same site on the male in each photo). It was not clear whether the movement of the adminiculum was passive (with its tip moving dorsally due to movement of the anterior wall of the genital chamber where its tip presumably made contact), or whether it was due to active pushing by the male (or both) (see text). (**d**) This schematic view summarizes changes in the positions of the male and structures during a squeeze (dashed lines 0.43 s previous). (**e**) Temporal sequence of periodic squeezes over a period of 14 s.

The adminiculum was mostly motionless, pressing against the membranous anterior wall of the female genital chamber (Figure 4c). The small portion of the intromittent organ that was visible was also motionless. The adminiculum briefly pressed strongly against the anterior wall, however, during two or three brief bursts of several strong thrusting movements of the male’s hypopygium. Each burst of thrusting lasted only a second or so. Each thrust caused the female sternite 8 to move dorsally, especially at its anterior end. It was not clear whether the movement of the adminiculum during a thrust was passive (with its tip moving dorsally due to movement of the anterior wall of the genital chamber where its tip presumably made contact), or whether it was due to active pushing by the male (or both). The inclination of the sclerite indicated by the red arrow in Figure 7b suggests an active pushing movement by the male; the dorsal elevation of the base of tergite 10 in this same image suggests a movement by the female. Apparently, the tip of the intromittent organ did not emerge from the female between the thrusts, because the male did not repeatedly probe the wall as might have been expected if the tip had been withdrawn and then reinserted. 

Outer gonostyle

Rapid brushing movements of the outer gonostyle caused the long setae on its margins to sweep back and forth across the hypovalve (Figure 6a). In one video sequence with a favorable angle of view, it was clear that the tips of these setae contacted the hypovalve during these brushing movements. There were three patterns of brushing movements. During one period of the copulation, the hypovalve was immobile while the outer gonostyle repeatedly made only a single brief anterior brushing movement that lasted approximately 0.03 s followed immediately by a posterior approximately 0.03 s movement (Figure 6c). During two later bouts (about 90 and 120 min after the observations began), the outer gonostyle instead made short bursts of multiple brushing movements (Figure 6a,d). Each of these rapid back-and-forth brushing movements was followed by a short period of immobility and then by another back-and-forth brushing movement. Early in one bout of this pattern of brushing, each series of three to nine quick single brushing movements was followed by an anterior movement of the female hypovalve that caused the outer gonostyle setae to brush across the hypovalve (Figure 6b). Later in this bout the hypovalve movements ceased, the rate of brushing gradually slowed (Figure 6d), and the amplitudes of the brushing movements decreased. The longest bout of repeated brushing lasted an estimated 30 to 120 s. 

A third pattern of brushing movements occurred during two periods during which the female made repeated “squeezing” movements with her cerci and hypovalves (Figure 7; see below). In these cases, the outer gonostyle made only a single, short (0:03 s) anteriorly directed brushing movement against the immobile hypovalve; in at least 8 of 10 cases this male movement was followed immediately (in the next frame of the 30 fps video) by the initiation of a female “squeeze”(see below). 

Tergites 7 and 8

**Figure 8:**
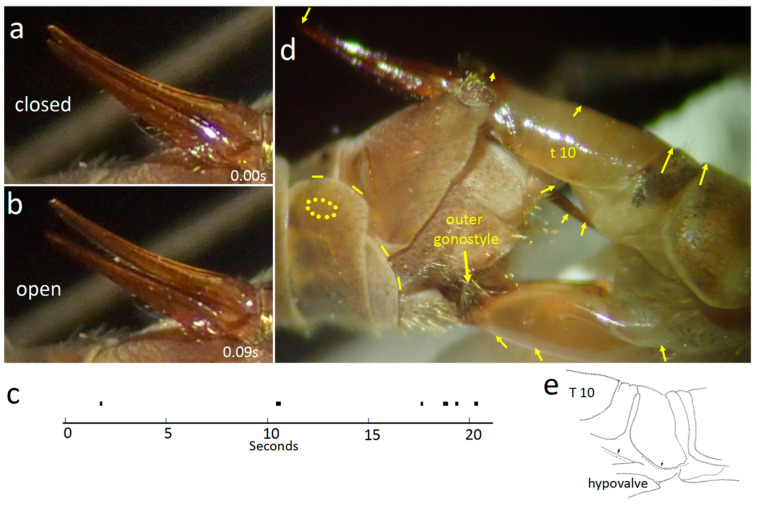
(**a**,**b**) The cerci of *T*. (*T*.) *colei* were more or less closed most of the time, but occasionally one or both opened laterally for a fraction of a second. (**c**) Temporal pattern of periods during which the cerci were open. (**d**) Different types of movements mentioned in the text are indicated schematically. The oval space marked with dots is the area in which the cuticle pulsed rhythmically; the orientations and lengths of the yellow arrows indicate the different directions and distances that different parts of the male and female moved during a weak squeeze; the yellow lines at the edges of male sclerites indicate their rhythmic movements relative to other sclerites. (**e**) The dashed lines indicate the displacements of female structures during a “twitch”(0.07 s later).

During one period 30–60 min after observations began, the posterior edge of the lateral and dorsal portions of male tergites 7 and 8 (which did not contact the female) slid rhythmically back and forth (yellow lines in Figure 8d) fourteen times during a period of 11 s). This movement did not occur either earlier or later in the copulation. Earlier in the copulation, a central portion of male tergite 7 buckled rhythmically (29 times in 24.5 s) (Figure 8d), suggesting that a muscle attached to its inner surface (perhaps the semen pump?) [36] was contracting rhythmically.

Appendage of sternum 9

**Figure 9:**
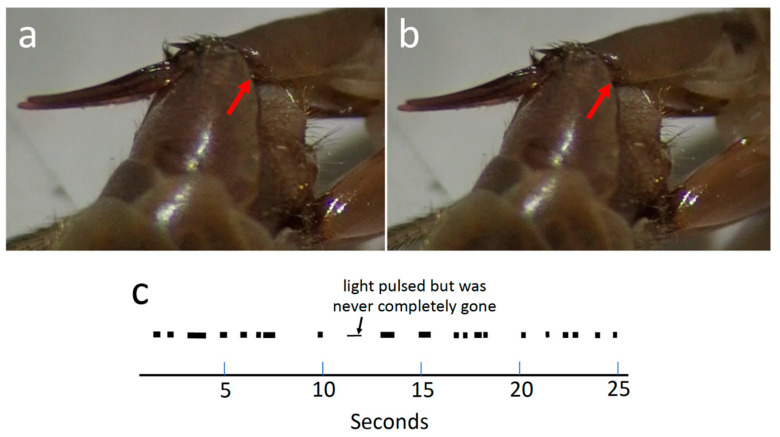
(**a**,**b**) Frames from a video of *T*. (*T*.) *colei* 0.2 s apart; the bright reflected light present on the undersurface of tergite 10 (red arrow) in the first frame has disappeared in the second (red arrow in (**b**)). (**c**) Temporal pattern with which this light was present during 25 s of one recording.

The appendage of male sternum 9 (A9S) was hidden from view under the female stalk, but there was a hint that its plate-like ventral lobe made rhythmic movements. In one video recording lasting 25 s, a bright spot, perhaps resulting from movements of A9S, appeared and disappeared rhythmically on the shiny lower surface of the female tergite 10, just basal to the lower edge of the male sternite 9 (Figure 9a,b). The mean duration of each shine was 0.35 ± 0.20 s (N = 21), and the mean time between initiations of shines was 1.11 ± 0.72 s (Figure 9c). None of the other visible portions of either the male or the female genitalia moved in coordination with this rhythm (most were immobile). As noted above, each ventral lobe of the appendage had long setae on its distal surface (Figure 2b), and the positions of the appendages suggest that they (or their setae) may have contacted the dorsal wall of the female’s genital chamber. 

Male structures that contacted the female but did not move independently

The lateral lobes of male sternite 8, their long, stiff, medially directed setae, and the nearby sub-median processes were all immobile with respect to other male genital structures. The male’s inner gonostyles, which probably meshed with the distal ends of the apparently immobile female hypovalves, were not visible. 

#### 3.1.5. Movements of Female Structures

“Twitches” of tergites and membranes

The distal portion of the female’s abdomen “twitched” and “pulsed” nearly continuously during the entire three hours of observation. Repeated movements included apparent small, brief movements of the base of the hypovalve toward the posterior margin of tergite 9, shortening of the distances between tergites 8, 9, and 10, brief movements of tergite 8 away from tergite 7, and brief expansions of the intersegmental membrane below tergites 8 and 9 (Figure 7e). 

“Squeezes” 

During the last 30–60 min of the copulation the female performed at least three bouts of large “squeezing” movements, in which the tips of her hypovalves moved distally and her cerci and stalk moved basally and dorsally (Figure 7). The mean duration of each of nine consecutive squeezes was 0.16 ± 0.07 s, with a mean of 1.59 ± 0.44 s between them. During a squeeze, the distance between the stem and the hypovalves decreased, but they did not touch each other or any other central object, so the term “squeeze” is not to be taken literally. The male’s adminiculum moved during each female squeezing movement, and its pressure against the anterior wall of the female’s genital chamber probably changed. It was not clear, however, whether it moved due to force applied by the male or by the female (or both). Some squeezes resulted in small dorsal movements of the distal portion of the female hypovalve, causing the long setae of the male’s outer gonostyle to move across the surface of the hypovalve (Figure 6b).

Cerci

Occasionally, one or both cerci opened briefly (for a mean of 0.19 ± 0.05 s; N = 13) and then closed (Figure 8a,b). The membrane ventral to female tergites 7 and 8 often inflated slightly as the cercus opened. Cercus movements caused the ventral surfaces of the basal portions of the female’s cerci to slide across the smooth surface of male sternite 8 between the submedial processes. The dorsal surfaces of the cerci may have also brushed against the medially directed setae on the lateral lobes of the male’s sternum 8 (Figure 5a). The female’s cercus movements may have also caused the lateral lobes and submedial processes of the male sternite 8 to move against the membranes where her cerci articulated with tergite 10. 

### 3.2. Genital Morphology and Behavior of T. (L.) translucida

**Figure 10:**
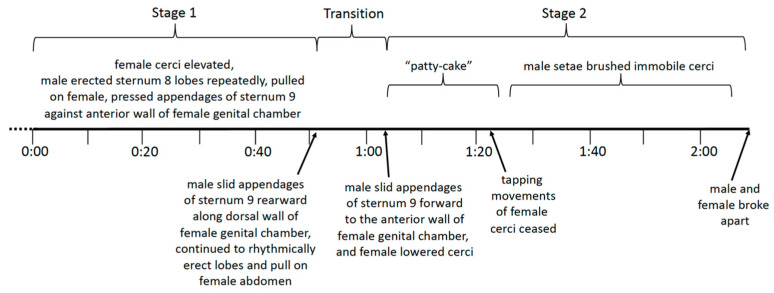
The sequence of major events in the video recording of the copulation of *T*. (*L*.) *translucida*. Details of the behavior are described in the text.

Two stages of copulation, defined by differences in both positions and behavior, will be described separately. Stage 1 lasted for the first 51 s after the beginning of the video recording; it was followed by an approximately 12 s transition and then stage 2, which lasted 140 s until the flies separated (Figure 10).

#### 3.2.1. Female Morphology and Behavior

**Figure 11:**
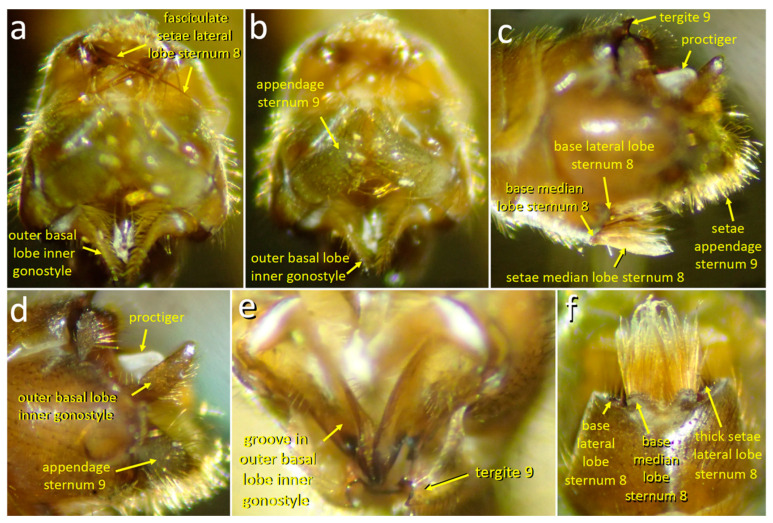
Posterior (**a**,**b**), lateral (**c**,**d**), dorsal (**e**), and posterior-ventral (**f**) views of the male genitalia of *T*. (*L*.) *translucida* illustrate structures mentioned in the text.

The basic female genital morphology of *T*. (*L*.) *translucida* is similar to that of *T*. (*T*.) *colei*. On the female’s dorsal side, the cerci articulate basally near the distal tip of tergite 10; ventrally, a pair of long hypovalves articulate basally on sternum 8, lateral to the membranous anterior wall of the genital chamber. During both stages 1 and 2 of copulation, the cerci and the hypovalves were spread apart dorso-ventrally as in *T*. (*T*.) *colei*, exposing the genital chamber between the two. The tips of the hypovalves pressed against the male’s dorsal genitalia, but the angle of viewing and the illumination made it impossible to discern details. Presumably, the tips of the hypovalves fit into the grooves in the basal portions of the male’s gonostyles and under the male’s ninth tergite (Figure 11e).

**Figure 12:**
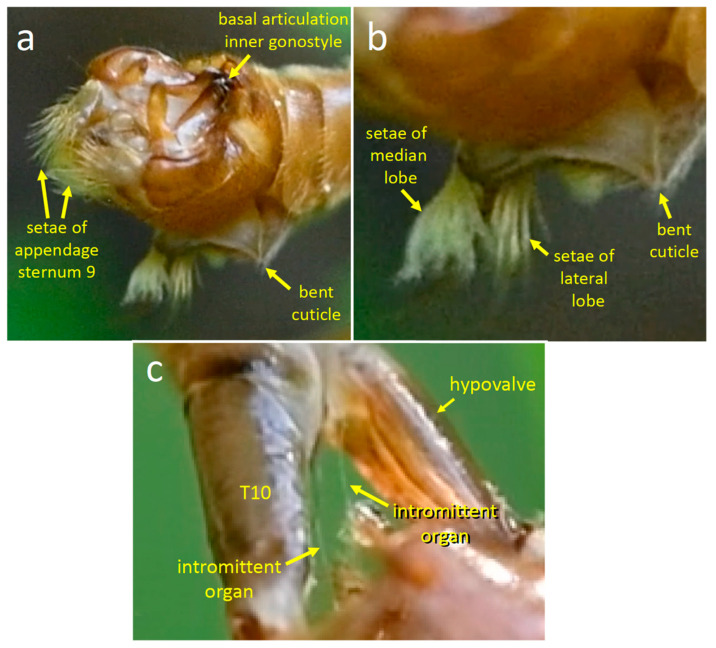
Images of *T*. (*L*.) *translucida* from video recordings made immediately following the end of copulation (**a**,**b**) illustrate the erection of the setae of the lateral and median lobes of sternite 8. The apparent intromittent organ (two thin white lines in (**c**); perhaps it was looped or folded) was revealed as the male’s genitalia pulled partially away from the female during the transition between stage 1 and stage 2.

**Figure 13:**
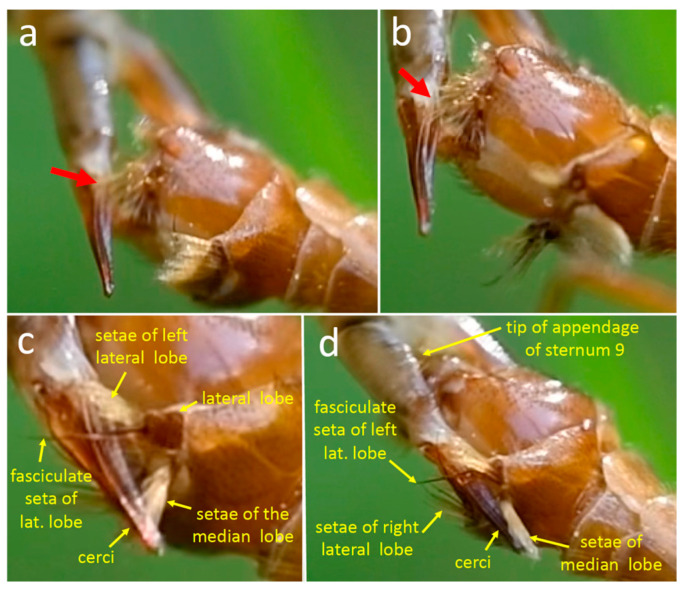
Stages of the transition between stage 1 and 2 in *T*. (*L*.) *translucida*. (**a**,**b**) The robust setae on the appendages of sternum 9 (red arrows) rubbed on the dorsal wall of the female’s genital chamber during the seconds preceding the lowering of the cerci. (**c**,**d**) During the first major contact between the female’s recently lowered cerci and the setae on lateral and median lobes of sternum 8 of the male, the fasciculate seta lay across and “embraced” the basal portion of the female’s cerci.

During stage 1 the stalk was flexed dorsally and remained immobile (Figure 12c and Figure 13a,b). It was lowered at the end of the transition between stage 1 and stage 2 so that its long axis was nearly parallel to that of the hypovalves, where it remained during stage 2 (Figure 13c,d). The cerci repeatedly tapped ventrally against the setae on the median lobe of the male’s sternite 8 during the first portion of stage 2 (below). There were no female movements that resembled the twitching, squeezing, and cercus opening movements of female *T*. (*T*.) *colei*.

#### 3.2.2. Male Morphology and Behavior during Copulation

The male’s abdomen was twisted 180° during the entire copulation so his ventral genital structures contacted the dorsal genitalia of the female and his dorsal genital structures contacted her ventral genitalia. All three pairs of flies were already coupled when they were discovered. The pair in the video recording was in stage 1 and transitioned to stage 2 while the two pairs in the still photographs were in stage 2. The earliest stages of coupling thus remain unknown. The mentions in the descriptions below of “modified” setae without any further specifications refer to tufts of long, yellow setae on the lateral and median lobes of sternite 8.

#### 3.2.3. Morphology

**Figure 14:**
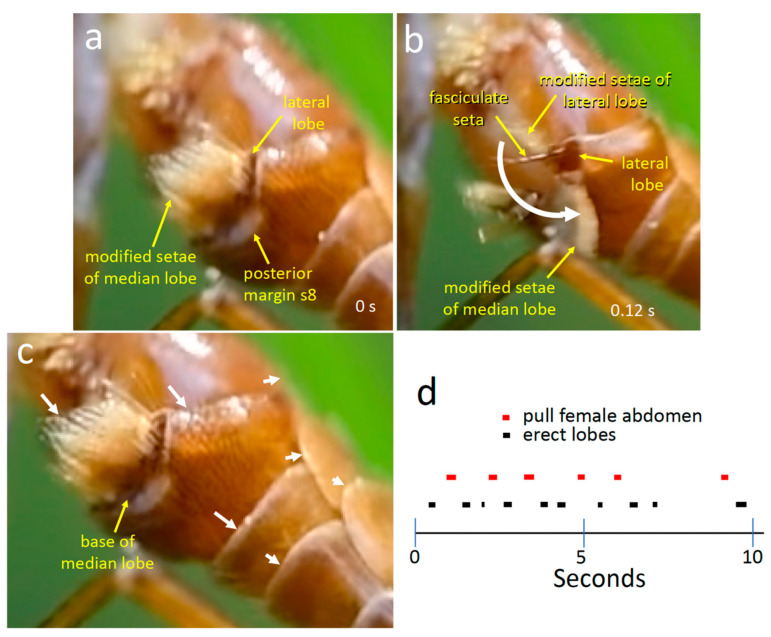
(**a**,**b**) Frames from a video recording of *T*. (*L*.) *translucida* illustrate (in dorso-lateral and slightly anterior views) the movements of the median and lateral lobes of sternum 8 during stage 1. The lobes were at rest in (**a**) and erected in (**b**) (the modified setae of the lateral lobes were moving rapidly in (**b**) and are blurred). The white arrow in (**b**) indicates the path of the modified setae of the median lobe. (**c**) This frame from a video recording illustrates a pull and twist movement by the male (arrows mark movements from the previous frame). (**d**) The rhythms of erecting the lobes and pulling on the female abdomen are shown graphically. There was no overlap between the periods of erections (thick black lines) and pulls (thick red lines).

**Figure 15:**
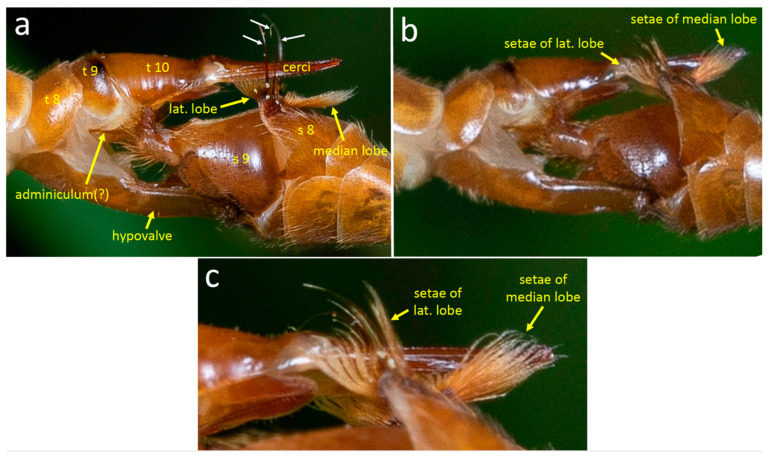
During stage 2 of copulation, the setae on the lobes of the male sternite 8 (s8) of *T*. (*L*.) *translucida* were repeatedly moved from their resting positions (**a**) to contact the female cerci and their basal articulations with female tergite 10 (t10) (**b**,**c**) (the female is on the left, the male on the right). In a, the white arrows mark fasciculate setae, one on the near side lateral lobe and one on the far side; “s” indicate sternites of the male; “t” indicate tergites of female; and the modified setae of the median lobe (“med. lobe”) and lateral lobe (“lat. lobe”) are labeled. (**b**,**c**) These frames from a video recording illustrate how the setae of the two lobes were deflected by and enveloped the female’s cerci when they tapped and rubbed against them.

**Figure 16:**
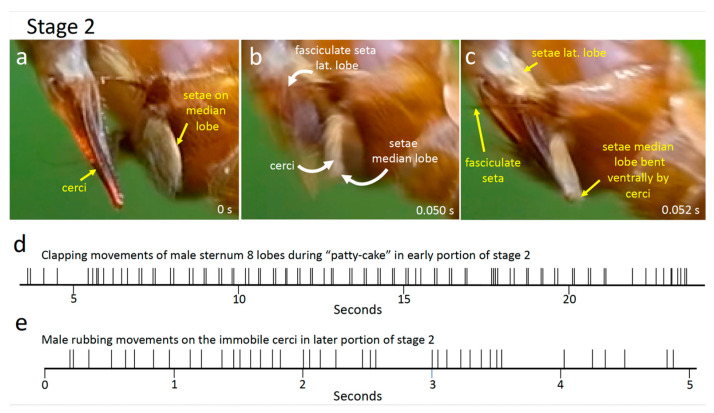
(**a**–**c**) Frames of a 60 fps video recording of *T*. (*L*.) *translucida* illustrate tapping and mutual male and female “patty-cake” movements early in stage 2. The elapsed times after the first frame are at the lower right (in seconds). Both the male lobes and the female cerci were more or less immobile for two frames (0.033 s) following (**a**); then they moved toward each other in the next frame (**b**); in the following frame, (**c**) the cerci continued their ventral movement, bending back some of the setae, and the setae on the lateral lobe tapped the base of the cerci. The white arrows in (**b**) show the directions moved by the cerci, the tips of the median lobe setae, and the fasciculate seta of the lateral lobe (**d**) The temporal pattern of the “patty-cake” movements of the cerci and the modified setae on the lateral and median lobes of sternite 8 early in stage 2. (**e**) The temporal pattern of rubbing movements by the male setae while they made sustained contact with the female’s immobile cerci late in stage 2.

The posterior margin of male sternite 8 is indented centrally and bears a pair of median lobes and a pair of lateral lobes (Figure 11c,f, Figure 12a, Figure 13d, Figure 14a and Figure 15a). Sternite 8 extends laterally on each side to a single point, and the lateral lobe is just posterior to this point (Figure 13d and Figure 14b). The lateral lobes were nearly completely hidden when at rest (Figure 11c,f), and were exposed only during copulation (Figure 13, Figure 14 and Figure 15). Each lateral lobe includes a planar patch of cuticle (Figure 13d, Figure 14b and Figure 16a), two long, dark, thick, curved “fasciculate” setae (Figure 14b and Figure 15a) that cross when at rest (Figure 11a), a dense tuft of long yellow setae, and a few short, dark, thick setae at their base (Figure 11f and Figure 15a–c).

The median lobes of the male sternite 8 lie just posterior to the central indentation in the rear margin of the sternite, separated from it by only a narrow membrane (Figure 11f). Each median lobe forms an approximate semicircle of cuticle and the two semicircles nearly contact each other (Figure 11f). Each median lobe bears a large, dense brush of long yellow setae (Figure 11f). At least some of the modified setae on the lateral lobes differ from other sharply pointed, yellow cylindrical setae on the lateral lobes in being somewhat flattened, bent irregularly at small angles, and kinky near their tips (Figure 11f and Figure 15c).

The pair of appendages of tergite 9 also bear dense arrays of setae on their distal surfaces and small spines that extend posteriorly (Figure 11c,d). The posterior margin of the male’s tergite 9 has a single, deep indentation (Figure 11f). The inner gonostyle is “V”-shaped and articulates basally against the posterior margin of tergite 8 (Figure 12a). The basal portion of the gonostyle lacks long setae but has a complexly contoured inner groove (Figure 11e and Figure 12a). The more distal outer basal lobe of the gonostyle has a flattened, paddle-like shape whose medial surface is covered with strong setae (Figure 11a).

#### 3.2.4. Stage 1

The distal portions of the female’s cerci and stalk were held immobile, lifted away from the male (Figure 12c and Figure 13a,b), and the tips of her hypovalves pressed against the dorsal portion of the male’s hypopygium (Figure 13a,b). The longitudinal axis of the female’s stalk made an angle of approximately 30° with the longitudinal axis of her hypovalves (Figure 13a).

The male’s ventral side

At rest during stage 1, the long yellow setae of the median and lateral lobes of the eighth sternum were directed ventro-posteriorly, approximately parallel to the longitudinal axis of the male’s abdomen (Figure 11c). The lateral lobes had similar orientations but were directed more medially, with their fasciculate setae crossing (Figure 11a). 

Both the lateral and median lobes of sternum 8 made large, repeated movements during stage 1, moving rapidly and simultaneously to “erect” positions during the space of about 0.1–0.12 s and then returning to their resting positions (Figure 14a,b and Figure 15a–c). The mean time from the beginning to the end of an erection movement was 0.20 ± 0.05 s (N = 40) (Figure 14d). The long yellow setae of the median lobe moved approximately 90–120^o^ in an antero-ventral direction to make an angle of approximately 45° with the posterior-to-anterior longitudinal axis of the abdomen (Figure 14b). The modified setae on each lateral lobe were spread in the erect position and were directed more anteriorly (Figure 14b and Figure 15b,c). The most rapid part of the erection movement was the movement back to the resting position.

Usually, all four lobes were erected simultaneously; the two median lobes always moved simultaneously, but the lateral lobes sometimes moved independently of each other and of the median lobes. The modified setae on the lobes did not contact the female’s elevated cerci (Figure 13a,b).

The mechanism used to erect the lobes was unclear. It appeared that deformation of the cuticle was involved, because each erection was accompanied by a sharp ventral flexion of the posterior margin of sternite 8 (Figure 12a,b and Figure 14b) and sternites 6–8 moved anteriorly with each swing. 

Brief pulling movements (Figure 14c,d) that forcefully extended the female’s abdomen rearward by pulling on her hypovalves were interspersed with erections of the lobes (Figure 14b). The male pulled by withdrawing his hypopygium and abdominal segments 6 and 7 (moving them toward more anterior portions of his body), and by bending his hypopygium slightly laterally (Figure 14c). The mean duration of a pull was 0.27 ± 0.06 s (N = 22), while the mean and median times between pull initiations were 1.87 ± 1.45 s and 1.34 s, respectively (N = 21). Pulling always occurred in lapses between erections of the lobes, but there was no other obvious coordination between the two types of movement (Figure 14d).

The robust setae on the distal ends of the tergite 9 appendages (Figure 11c,d) made sustained contact with the membranous dorsal wall of the female’s genital chamber during stage 1 (Figure 13a–c). Most of the time these appendages did not move independently of the rest of the male’s genitalia, but occasionally they made minor, apparently passive movements against the wall when the hypopygium moved dorsally. This description may underestimate of the mobility of the appendages, however, as in several cases during stage 1 the bristles at the tip of the appendage were in good focus, making it possible to see that all or a substantial subset of them made apparently independent, simultaneous movements.

The male’s dorsal side

No independent movements of the male dorsal genital structures were seen during copulation, but the angle of view and lighting were such that minor movements (if they occurred) could have gone unnoticed (the inner gonostyles made repeated, active, coordinated movements immediately following copulation—see below).

#### 3.2.5. The Transition to Stage 2

The approximately 12 s transition from stage 1 to stage 2 began about 0.3 s after the end of an erection of the lobes of sternum 8, when the setose distal portions of the appendages of sternum 9 moved to press against the anterior wall of the genital chamber. About 0.2 s later, the tips of the appendages scraped posteriorly along the dorsal wall of the genital chamber (Figure 13a,b) while the entire hypopygium pulled away from the anterior wall of the female’s genital chamber, and two thin structures (presumably portions of the intromittent organ) were revealed stretching from the male to the female (Figure 12c). Meanwhile, the dorsal portions of the male’s genitalia remained in contact with the tips of the female’s hypovalves.

The male immediately began a series of erections of the lobes of sternum 8, and in addition, pulled repeatedly on the female’s abdomen with movements of his hypopygium (Figure 14a–d). These hypopygium movements caused the setose tips of the appendages of his sternum 9 to scrape against the dorsal wall of the female’s genital chamber (Figure 13b). Contact with the wall was verified in several cases, as the movements of the appendages resulted in dorsal movements of the cerci.

Three major position changes occurred at the end of the transition. The female lowered her cerci substantially so that they were more nearly parallel to her hypovalves (Figure 13c). The distal ends of the appendages of sternum 9 moved anteriorly along the dorsal wall of the genital chamber to press against the chamber’s anterior wall (Figure 13c) (visibility of the appendage tips was limited, but it was clear that they flexed ventrally at least once during this repositioning, rubbing across the anterior wall of the genital chamber). Additionally, the male’s lateral and median lobes ceased returning to their previous resting positions and instead spent most of the time flexed anteriorly (Figure 15a). 

In summary, the transition began when the male pulled his hypopygium temporarily away from the anterior wall of the female’s genital chamber; his intromittent organ nevertheless apparently remained inserted in the female, and the distal portions of her hypovalves remained in contact with his dorsal genitalia. The setae on the male’s sternum 9 appendages rubbed across the dorsal surface of the female’s genital chamber during this withdrawal and during the following 10 s while the male alternately pulled the female’s genitalia rearward and erected his sternum 8 lobes. Finally, his sternum 9 appendages moved anteriorly to again press their setose tips against the anterior walls of her genital chamber, and the female immediately lowered her cerci. In this position, the female’s cerci were in range of contact with the male setae on his lateral and median lobes when they were erected. The lowering of the female’s cerci was not in any way forced mechanically by the male. 

#### 3.2.6. Stage 2

During the early portions of stage 2, the female’s cerci and the brushes of setae on the male’s lateral and median lobes moved repeatedly and nearly simultaneously toward each other with quick, highly stereotyped “patty-cake” movements to strike each other. The brushes contacted and enveloped the cerci and their basal articulations each time they came together (Figure 15b,c and Figure 16a–c). In 21 cases, the male and female movements began simultaneously, and in 56 others, the male lobes began to move one frame earlier than the female cerci (it is possible that the only moderate speed of the video recording (0.017 s/frame) was not sufficiently rapid to always register differences in initiation times and that all male movements began earlier than those of the female). Usually, both the lateral and the median lobe setae of the male moved synchronously, but in a few cases the lateral brushes started one frame earlier. 

With each contact movement, the cerci and tergite 10 of the female swung even farther ventrally in the frame or frames that followed initial contact, causing some of the male’s median lobe setae to bend dorsally (Figure 16c). In the next two or three frames, the cerci reversed direction and swung away (dorsally), but the setae continued to make contact with them; in subsequent frames a space opened up between them as the cerci continued to move dorsally and the setae reversed direction and moved back to their original positions. In at least some cases the long thick, fasciculate seta on the lateral lobe of sternum 8 approached and may have contacted the dorsal surfaces of the cerci (the viewing angles precluded certainty on this point); in other cases, however, these spines clearly did not contact the cerci (Figure 16c). “Patty-cake” movements of the male’s sternite 8 lobes and the female’s cerci were repeated 77 times in the space of about 18.5 s (Figure 16d). 

The male’s sternum 9 appendages also moved repeatedly during “patty-cake” behavior, flexing dorsally with respect to the female, and scraping posteriorly across the dorsal wall of her genital chamber before returning to press on the anterior wall. These small movements were less easily observed but appeared not to be tightly coordinated with the “patty-cake” movements. 

In the video recording of the transition between stage 1 and stage 2, the tip of the adminiculum was visible but did not reach the anterior wall of the genital chamber. The angle of view precluded observation of this detail during stage 2. In a still photograph of a copulating pair in stage 2, however, the adminiculum extended to the anterior wall of the female’s genital chamber (Figure 15a).

The ventral movements of the female cerci gradually became smaller toward the end of the “patty-cake” period and eventually, about 19 s after stage 2 had begun, they ceased completely. Within 0.5 s, the movements of the male’s lateral and median lobes began to be smaller and more rapid. For about 1 s, the setae on these lobes remained out of contact with the cerci, but the male then pressed them against the cerci where they rhythmically brushed or quivered against the cerci without breaking contact for about 18 s. During one lapse of 4.7 s with good visibility, the setae of the median lobe made at least 39 brushing movements on the cerci. These brushing movements were thus on the order of twice as frequent as the patty-cake movements.

Finally, the female raised her cerci dorsally, pulling them out of contact with the male. The male’s lobes tapped about five times in the air without contacting her, and he then began to pull his sternum 9 appendages and the rest of his hypopygium posteriorly (no intromittent organ was seen, but visibility was not optimal). About 1.5 s later the tips of her hypovalves began to slip away from the dorsal portion of the male’s abdomen and both flies pulled their abdomens away rapidly, just over 60 s after the beginning of stage 2.

#### 3.2.7. Male Genital Behavior Following Copulation

The male’s genitalia continued to move during the 128 s that were recorded in dorsal view immediately following copulation. The biological significance of these movements is unclear (perhaps the copulation was interrupted prematurely?), but they revealed several behavioral capabilities that were not obvious during copulation. 

**Figure 17:**
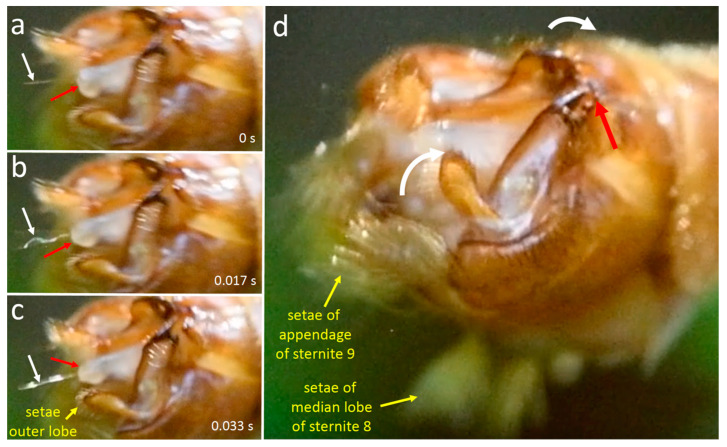
Genital movements of a male *T*. (*L*.) *translucida* soon after copulation ended illustrate (**a**–**c**) how the intromittent organ (white arrows) bent temporarily while being extended (**b**) and the tip of the proctiger changed form (red arrows). (**d**) shows the anterior rotation of the hypopygium (upper white curved arrow) and the partial rotation and lifting of the inner gonostyle (lower white curved arrow) when it moved anteriorly at the articulation with tergite 8 (red arrow). The lobes of sternite 8 were also erected at the same time.

The entire hypopygium repeatedly swung dorsally, as if pivoting in a socket at the posterior end of segment 7. During each dorsal swing or pivot of the hypopygium, the lobes of sternum 8 spread apart (Figure 17d) (only the setae of one set of lobes were visible; presumably both lobes were spread each time, as occurs during copulation). The outer basal lobes of the inner gonostyles (which were out of sight during copulation) were spread apart at the beginning of some dorsal swings and then closed toward each other while the hypopygium swung dorsally. In at least some cases, the inner gonostyles twisted laterally as they spread apart, causing the outer basal lobes to spread apart laterally (compare Figure 17a–c with Figure 17d). When these lobes closed toward each other, the gonostyle swung dorsally in a movement (white arrows in Figure 17d) hinged where the inner gonostyle articulated at the rear margin of tergite 8. In some cases, the inner gonostyles also rotated to move medially while they moved dorsally, causing the setae on the flat outer basal lobes to contact each other. In other cases, these gonostyles remained more or less closed as the hypopygium swung dorsally or spread apart while the hypopygium was not moving. Similar closing movements of the inner gonostyles, if they occur during copulation, would presumably cause these setae to press against the distal portions of the female hypovalves.

Another male genital movement seen only following copulation was the repeated extension and retraction of the thin intromittent organ (Figure 17a–c). It was evidently relatively flexible, as in one frame of the video it bent substantially while being extended (Figure 17b). The intromittent organ was only occasionally in focus and appropriately illuminated, so further details of its movements and possible coordination with other genital movements were uncertain. 

## 4. Discussion

In the discussion below, acronyms are used to indicate different hypotheses to explain genital evolution: SC for sperm competition; CFC for cryptic female choice; SAC for sexually antagonistic coevolution; and SI for species isolation.

### 4.1. Apparent Stimulation Functions for Male Genitalia

#### 4.1.1. *T. (T.) colei*

Several details suggest that the brushing movements of the long setae of the outer gonostyle of male *T*. (*T*.) *colei* function to stimulate the female’s hypovalve. The long, curved forms of these setae, their locations on the edge of the outer gonostyle, and the position of the gonostyle next to an expanse of hypovalve surface are appropriate to produce a wide area of contact with the female hypovalve. The fact that females immediately responded to some brushing movements with squeezes indicates that the female was able to sense the male’s brushing movements. Alternative functions for these setae, such as physical clamping or holding the female, are ruled out by the lack of appropriate mechanical properties: the setae cannot exercise appreciable mechanical force. Their movements were also ill-designed to produce mechanical force on the female. Conspicuous by their absence are any female structures or behaviors that could impede male contact or brushing, as predicted to be common by the mechanical versions of SI and CFC hypotheses and both mechanical and stimulation versions of the SAC hypothesis. 

A second set of male structures, the lateral lobes of sternum 8 and their tight bundles of stiff, medially directed long setae, contact and thus inevitably stimulate the dorsal bases of the female’s cerci and their articulations with her tergite 10 (assuming there are stretch sensors present in the membrane). Here again, possible female defenses against such stimulation are lacking. The basal articulation of the cerci of *T*. (*T*.) *colei* shows no modification that could physically impede its being clamped by the lateral lobes of male sternite 8.

The prolonged contact of the tip of the adminiculum with the female genital chamber wall and its periodic thrusts against it also represent possibly stimulatory behavior. The distal surface of the adminiculum presumably also contacts the genital chamber wall in many other tipulids during the process of inserting the intromittent organ into the female’s bursa. Tangelder [21] described a similar position in *Nephrotoma* spp. Apparently, no movements of the adminiculum have been described in other tipulids. The sustained contact of the adminiculum with the anterior wall of the female genital chamber, combined with the fact that adminiculum morphology often diverges rapidly and provides good species characters [27,36], fit the stimulation versions of the SI, SAC, and CFC hypotheses. The soft, membranous nature of the wall, in contrast, does not fit the mechanical versions of these hypotheses. 

Some other possibly stimulatory male genital structures of *T*. (*T*.) *colei*) (e.g., the tuft of long setae on the tip of the distal lobe of the inner gonostyle (also called the crest) (Figure 1a,d) were never seen to contact the female. The early stages of copulation were not observed, however, so the possibility that they are used as stimulatory structures cannot be discarded.

#### 4.1.2. *T. (L.) translucida*

The movements and morphological designs of *T*. (*L*.) *translucida* genitalia also suggest that several male structures function to stimulate the female. The clearest evidence is from the median and lateral lobes of sternum 8. Their long, abundant setae are located precisely on the portions of the lobes that contact the female: the modified forms of the especially dense setae with flattened, kinked forms and crinkled tips are appropriate to increase even further their points of contact with the female. The tapping, brushing, and vibrating movements of the lobes and their setae are also appropriate to increase stimulation by avoiding habituation and neural adaptation by female touch receptors [39,40]. Alternative functions for the setae and their movements, such as forcefully manipulating the female, are confidently ruled out by their mechanical properties, which are inappropriate to exercise appreciable force on the female. The lack of defensive female structures on the cerci that would impede contact with these male setae does not fit either mechanical versions of the SI, CFC, or SAC hypotheses or the stimulatory version of SAC. 

Several details indicate that the dense setae on the tips of the appendages of sternum 9 also function to stimulate the female. These setae repeatedly scraped against the membranous anterior and dorsal walls of the female’s genital chamber, especially during the transition between stages 1 and 2 and the early portion of stage 2. This scraping was produced both by movements of the entire hypopygium and by small amplitude movements of the appendages themselves. The simultaneous movements of strong setae while in contact with the anterior wall of the female genital chamber constitute another movement that is appropriately designed to stimulate the female and that has no other obvious function. The dense array of robust setae on the appendages are located on just those portions of the lobes that contact the female, so these different movements likely stimulate the female. Alternative functions for these setae and their movements, such as holding the female, are also confidently ruled out by their positions and mechanical properties. 

The strict coordination seen in the male’s behavior immediately following copulation between the erections of the lobes of sternum 8, the dorsal flexion of the hypopygium, and the spreading and closing movements of the gonostyli suggest that similar flexions of the gonostyli against the female’s hypovalves also occur during copulation. Such flexions could result in stimulation of the female. This is only speculation, however, since no movements were observed directly during copulation.

Neither the division of *T*. (*L*.) *translucida* copulation into two stages with different male and female positions and movements nor the precise coordination between male and female “patty-cake” movements has been reported for any other tipulid.

In sum, there are strong indications from both behavior and morphological design that at least one male genital structure in each species functions to stimulate the female. There are less definitive indications in both species that at least one other structure may also have this function. There are several possible payoffs to males from stimulating the female during copulation because the selection of several types could favor selective female responses to male stimulation. These include allowing sperm transfer and storage to occur, determining the male’s species identity, avoiding male manipulations that utilize sensory traps, improving offspring quality, and improving male–female coordination of reproductive processes (e.g., [3,41,42,43,44]). Additional data of this sort, as well as experimental modifications of male stimulation and/or female sensitivity to stimulation, would be needed to further test the possibility of stimulatory functions for male genitalia in these flies. 

### 4.2. Phylogenetic Considerations

*Tipula colei* in the subgenus *Triplicitipula* and *Tipula translucida* in the subgenus *Lunatipula* are in the *Vestiplex-Lunatipula* clade of *Tipula* that contains several other subgenera. A phylogeny of this clade, developed by Gelhaus [27] using primarily male and female genitalic characters, indicates that three of the structural systems of the male genitalia discussed in this paper are newly derived structures within this clade: (1) the distal lobe and specialized setae of the appendage of the ninth sternum (characters 13–17 of [27]), (2) the paired median lobes with hair brushes on the eighth sternum (character 33 of [27]) and (3) the lateral lobes and specialized fasciculate setae of the eighth sternum (characters 34–35 of [27]). In addition, the elaboration of a sclerotized ventral plate and lobes of the ninth tergum in the male (to presumably press around the female hypovalves) (characters 5–9 of [27]) and the development of a long, thin intromittent organ (character 36 of [27]) are also only found within this clade and are present in both species. Thus, some of the important male–female genitalic interactions in this clade that were observed in *T. colei* and *T. translucida* involved the use of newly derived and/or modified structures. 

The use of the male outer gonostyle and its long apical setae to brush the female hypovalves in *T. colei* is surprising because the outer gonostyle is progressively reduced in size and flattened within this clade. But when compared to the rest of *Tipula*, the setae near the apical tip of the outer gonostyle setae are longer, a novel feature that is appropriate for stimulating the female. An outer gonostyle with an anterior extension coupled with long setae is a derived feature in the subgenus *T*. (*Eremotipula*) [27] that may also function to stimulate the female. 

The use of derived traits to stimulate the female in the *Lunatipula*-*Vestiplex* clade is echoed in another subgenus of *Tipula, Bellardina* [35]. In *Bellardina* species, the outer gonostylus is large, thick, and complexly shaped, and there are large extensions of the ninth sternum that clasp the base of the female cerci, a defining feature of this subgeneric group that is derived with respect to other *Tipula* [24,45]. In this case, the male outer gonostyle stridulates against ridges on the male’s ninth sternite extensions during copulation [35], while the outer gonostyle setae in *colei* brush against the female hypovalves. The non-homologous structures of the male sternum 9 of *Bellardina* also differ: they hold the female cerci, while the appendages of the ninth sternum of *translucida* and perhaps also *colei* are pressed against the female genital wall between the cerci and hypovalves. 

### 4.3. Alternative Hypotheses Regarding Function

#### 4.3.1. Sperm Competition

The observations reported here, as well as those of other tipulid species [21,25,26,30,32,35], do not fit the sperm competition hypothesis for genital evolution. None of the male’s complex, species-specific genital structures ever enter the female’s body. They thus have no contact with the sperm of other males and are not able to remove or otherwise inactivate them without the mediation of the female (see discussion in [41] for distinguishing sperm competition from cryptic female choice). 

#### 4.3.2. Guide the Female

Tangelder [21] argued that modifications of male sternite 8 and tergite 9 function to orient the male and female to facilitate genital coupling. She mentioned, for instance, that they may “guide” the female cerci or the hypovalves, that “the spines [e.g., on sternite 9] are presumably sense-organs” (p. 143), and that elongate setae on sternites 8 and 9 in *Nephrotoma* and other Tipulidae function as sense organs to guide positioning of the female (or perhaps also the male) genitalia [21]. Several details argue, however, against this “guiding” hypothesis for the species and structures discussed in this study. 

In the first place, the movements of the male’s outer gonostyles in *T*. (*T*.) *colei* and of the male’s sternum 8 lobes and the sternum 9 appendages in *T*. (*L*.) *translucida* occurred long after the male and female genitalia were already coupled, when further guidance seems unnecessary. The rhythmic genital movements in both species are also unexplained by this hypothesis. The possibility remains that the guiding hypothesis is correct for structures that function during the early stages of coupling, especially when the male is twisting his abdomen 180^o^ to couple with the female, and the adminiculum may guide or support the intromittent organ. However, no observations are available to test these possibilities. In sum, the guiding function is unlikely for the morphology of several of the structures whose behavior is described here. 

#### 4.3.3. Apply Chemical Cues

Another possible interpretation is that the specialized male setae and their movements serve to disperse chemical signals onto the female, rather than to provide tactile cues. This possibility cannot be confidently dismissed, especially for *T*. (*L*.) *translucida*. Nevertheless, even if the chemical cue hypothesis is correct, the fact would remain that the male also produces tactile stimuli with his brushing movements. Both the tactile and the chemical possibilities are in accord with the basic hypothesis that male genital morphology and behavior function to stimulate females. 

#### 4.3.4. Other Functions That Are Ruled Out

The behaviors described here clearly contradict the mechanical versions of the SI, SAC, and CFC explanations of the evolution of these genital structures [3]. In addition, both the lack of plausible female defensive structures that could reduce male stimulation and the active female participation in events that increased rather than decreased her stimulation in both species (cercus spreading in *T*. (*T*.) *colei,* “patty-cake” behavior in *T*. (*L*.) *translucida*) argue against the stimulatory versions of both SI and SAC. Although female movements were not included in previous surveys of contact courtship behavior [46,47], it is reasonable to suppose that females may sometimes participate actively in sensing tactile stimuli from males. An equivalent behavior for visual courtship cues would be for a female to turn her head to look at a male’s display. Female movements of this sort could also function to communicate any one of several messages to the male [44]. If pre-copulatory filtering of heterospecific pairings occurs in the species of this study, as in *Tipula oleracea* [48], it would also argue against a species isolation function for the male genitalia.

### 4.4. Limitations of This Study

Only a single video recording was made of copulation in both *T*. (*T*.) *colei* and *T*. (*L*.) *translucida*, and all of the copulations observed had begun at unknown times previously. Nevertheless, several types of behavior were repeated frequently and rhythmically and were thus unlikely to be incidental occurrences. The descriptions here are more significant in a qualitative than in a quantitative sense. Hemmingsen, who had extensive experience with tipulid copulation behavior and its degree of variation, also utilized data from single copulations in several species [26,32].

The earliest stages of copulation, in which the male twists his abdomen and orients it appropriately to couple his genitalia with those of the female, have not yet been described carefully in the species of this study (or in any other tipulid). It seems likely that additional postures, sites of contact, and movements of the male and female genitalia occur during these stages. For instance, the rounded indentations in the rear margins of male tergite 9 in *T*. (*T*.) *colei* and *T*. (*L*.) *translucida* resemble the similarly rounded surfaces of the female hypovalves, but this male tergite was not observed to contact the female. The long fasciculate setae on the lateral lobes of sternite 8 of *T*. (*L*.) *translucida* (which could be seen only intermittently in the video recordings) could also have as yet undetermined roles.

More confident interpretations of stimulatory functions of genital morphology and behavior await further tests with experimental manipulations of male stimuli and/or female perception of these stimuli.

## Data Availability

Copies of the video recordings can be obtained from one of the authors (WGE) on request.
